# The effect of compliance to a Rigo System Cheneau brace and a specific exercise programme on idiopathic scoliosis curvature: a comparative study: SOSORT 2014 award winner

**DOI:** 10.1186/1748-7161-9-5

**Published:** 2014-05-30

**Authors:** LouAnn Rivett, Aimee Stewart, Joanne Potterton

**Affiliations:** 1 Physiotherapy Department, School of Therapeutic Sciences, Faculty of Health Sciences, University of the Witwatersrand, Johannesburg, South Africa

**Keywords:** Adolescent Idiopathic scoliosis, Spinal deformity, Spinal curvature, Compliance, Quality of life, Personality traits, RSC brace, Scoliosis exercise

## Abstract

**Background:**

There is controversy as to whether conservative management that includes wearing a brace and exercises is effective in stabilising idiopathic scoliosis curves. A brace only prevents progression of the curve and has been shown to have favourable outcomes when patients are compliant. So the aim of this study was to: determine the effect of compliance to the Rigo System Cheneau (RSC) brace and a specific exercise programme on Idiopathic Scoliosis curvature; and to compare the Quality of Life (QoL) and psychological traits of compliant and non compliant subjects.

**Methods:**

A pre/post test study design was used with a post study comparison between subjects who complied with the management and those who did not. Fifty one subjects, girls aged 12-16 years, Cobb angles 20-50 degrees participated in the study. Subjects were divided into two groups, according to their compliance, at the end of the study. The compliant group wore the brace 20 or more hours a day and exercised three or more times per week. The non-compliant group wore the brace less than 20 hours a day and exercised less than three times per week. Cobb angles, vertebral rotation, scoliometer readings, peak flow, quality of life and personality traits were compared between groups, using the student’s two sample t-test and an analysis of covariance.

**Results:**

The compliant group, wore the brace 21.5 hours per day and exercised four times a week, and significantly improved in all measures compared to non compliant subjects, who wore the brace 12 hours per day, exercised 1.7 times a week and significantly deteriorated (p < 0.0001). The major Cobb angles in the compliant group improved 10.19°(±5.5) and deteriorated 5.52°(±4.3) in the non compliant group (p < 0.0001). Compliant subjects had a significantly better QoL than the non compliant subjects (p = 0.001). The compliant group were significantly more emotionally mature, stable and realistic than the non compliant group (p = 0.03).

**Conclusions:**

Good compliance of the RSC brace and a specific exercise regime resulted in a significant improvement in curvatures, poor compliance resulted in progression/deterioration. A poorer QoL in the non compliant group possibly was caused by personality traits of the group, being more emotionally immature and unstable.

## Introduction

The cause of spinal deformity is a problematic developmental process, the outcome of which can be altered with appropriate intervention and there is some evidence that scoliosis is reversible [1-3]. Opinions vary about the efficiency of conservative treatment of scoliosis [4,5] with the efficiency of bracing continuing to be questioned [6-8]. The differences in the results of conservative treatment occur because of: a lack of standardisation of protocols and data analysis, a lack of reliable information about the natural history of untreated scoliosis [9]; as well as variations in brace type and lack of standardisation of application [10]. There are still many unknowns about brace treatment, which are required to determine its effectiveness [8] for example: the number of hours a day the brace should be worn to achieve optimum results [11]; what is the best weaning protocol and when weaning should start [8,12]; and how much “in brace” correction is needed to obtain good results. The primary aim of conservative scoliosis management is to stop curvature progression [5,8,13-15] and thus to avoid surgery [16]. Other important aims are the improvement of pulmonary function, treatment of pain, improvement of the aesthetic appearance [17,18], postural balance and reduction of psychological distress.

High correction bracing has been shown to have favourable outcomes when the patient is compliant [5,14,19-22]. Wearing the brace for 23 hours a day is significantly more successful than wearing the brace for between eight and 16 hours per day [10]. Longer hours of brace wear are associated with greater benefit [23]. Compliance and primary in- brace correction are the two most important variables associated with good brace outcomes [5,24,25].

There are differences of opinions as to whether exercise alone is a useful intervention for AIS [26-28]. Exercise has been shown to improve signs and symptoms of scoliosis without surgery [1,2,5,27,29,30] by maintaining the flexibility of the spine, which is important for rigid curves that progress rapidly. In a comprehensive systematic review of the literature, Negrini et al. [31], found that exercise reduces the curve progression rate and reduces brace prescription, with very specific exercises decreasing the progression of scoliosis to the point where a brace is not required [31]. Non-specific exercises are not effective in treating AIS [32]. The only randomised controlled trial (RCT) on exercise in AIS included 80 subjects, 15 (±4) years of age, with a 24° (±12°) Cobb angle. After six months of treatment, including gymnastic exercises, postural training and auto-correction, the Cobb angle in the exercise group improved 15°. Noteworthy here is that the mean Cobb angle was not large enough to brace and the study included only six months of treatment [33]. There is therefore very little evidence showing that conservative management on 25-50° curves improves Cobb angles.

The diagnosis and treatment of AIS can have significant psychological consequences for affected individuals [34] as the diagnosis of AIS comes at a time when concerns with appearance and bodily function are at their peak [4]. The impact of scoliosis is particularly marked if a brace is indicated as bracing for IS is a stressful experience [34,35] with psychological issues being blamed for the lack of compliance to wearing a brace [4,36]. Scoliosis is thus seen as a risk factor for impairment of quality of life (QoL) in adolescents [34,37].

Brace treatment can negatively affect health related quality of life (HRQoL) in scoliotic patients [38]. The SOSORT 2005 consensus paper, reported that only 1,48 percent of scoliosis studies included a measure of HRQoL [18]. Several reviews of the literature show that bracing does not affect the HRQoL in scoliotics [39] however some show that it does [34,40]. The Brace Questionnaire (BrQ) was designed to test HRQoL in brace treated scoliosis patients [38] and its use shows that by the end of conservative management, HRQoL deteriorates [41]. One possible explanation is the stress reported during conservative treatment with an orthotic device [35]. The impact of the brace on the self and body image is the main contributing factor to the stress [38]. Continuous monitoring of stress levels allows the possibility of modifying treatment in order to maintain good compliance [42].

Factors affecting compliance need to be identified. Poor compliance with wearing a brace is associated with poor QoL [43], which may relate to psychosocial coping mechanisms. Different personalities respond differently to a given situation [44]. Personality is the integrated and dynamic organisation of the individual’s psychic, social, moral and physical characteristics, as it obtains expression in the person’s interaction with the environment and with other people [45]. Carl Jung believed that personality traits were inborn, inherited genetically determined [46]. The High School Personality Questionnaire (HSPQ)(for ages 12-18 years) and the Sixteen Personality Factor Questionnaire (16PF)(for 18 years and older) give a valid and reliable picture of personality [47]. The HSPQ can be used to identify individuals with emotional and behavioural problems, to understand individuals and their problems better, to predict future school achievement, to promote pupils’ self-knowledge and to monitor personality growth [47]. The 14 personality traits have been shown to be good predictors of social, clinical, occupational and school behaviour [47,48]. See Table 1 below. The HSPQ and 16PF have been translated into over 35 languages and may be used by psychologists to predict the effectiveness of any therapeutic treatment.

**Table 1:** Fourteen primary factors of the high school personality questionnaire

**Factor**	**Low score description**	**Standard ten score (Sten) average**										**High score description**
		**1**	**2**	**3**	**4**	**5**	**6**	**7**	**8**	**9**	**10**	
A	Critical, reserved, cool											Warm, soft-hearted, participating, outgoing
B	Dull, less intelligent, concreteness											More intelligent, bright, abstract, thinking
C	Emotionally immature, unstable											Emotionally mature, stable, realistic
D	Deliberate, stodgy, placid, phlegmatic temperament											Unrestrained, nervous, excitability
E	Obedient, mild, dependant											Assertive, aggressive, rebellious, dominance
F	Sober, silent, serious											Happy-go-lucky, enthusiastic
G	Casual, quitting, undependable, opportunistic											Conscientious, preserving
H	Timid, threat-sensitive, shy											Venturesome, thick-skinned, social boldness
I	Practical, tough-minded											Tender minded, sensitive, protected,
J	Vigorous, goes readily with group, zestfulness											Individualistic, obstructive, reflective
O	Secure, resilient, confident											Discouraged, worrying, self-reproaching, prone to guilt feelings
Q^2^	Group follower, values social approval											Make own decisions, resourceful, self sufficiency
Q^3^	Careless, ignores standards, lax											Self-controlled, self-respecting
Q^4^	Relaxed, composed	1	2	3	4	5	6	7	8	9	10	Tense, driven, irritable

Long term studies on conservative therapies to stabilise or reverse scoliosis are required, before it progresses to lifelong difficulties. The aim of this study was to: determine the effect of compliance to the Rigo System Cheneau (RSC) brace and a specific exercise programme on IS curvature; and to compare the quality of life (QoL) and psychological traits of compliant and non- compliant subjects.

## Methods

### Study design and subjects

A pre test/post test study design was used with a post study comparison between subjects who complied with the management and those who did not. Ethical clearance was obtained from the Committee for Research on Human Subjects at the University of the Witwatersrand, (M060702). Subjects gave assent and parents signed informed consent before participating in the study.

Subjects were drawn from a private physiotherapy practice in, Johannesburg, South Africa. The inclusion criteria were: presence of idiopathic scoliosis, girls between the ages of 12 –16, Cobb angle between 20-50 degrees and no prior treatment. Exclusion Criteria were: other types of scoliosis, curves greater than 50 degrees, subjects who had previous surgery for scoliosis and previous treatment. Subjects were divided into two groups according to their compliance, which was recorded in their diaries after the brace and exercise interventions were complete and when weaning out of the brace had begun. The compliant group was defined as those subjects who wore the brace 20-23 hours a day and exercised three or more times per week. The non-compliant group was those subjects who wore the brace less than 20 hours a day and exercised less than three times per week. The study was terminated the day the subjects started weaning out of the brace. Weaning occurred when skeletal maturity was reached, that is, as close to Risser 5 as possible and subject’s height measurements had been static for at least six months.

Of primary interest was the change in Cobb angle and the power calculations were done for this parameter. A sample of 13 subjects per group had 90% power to detect a clinically relevant difference of 7% change in Cobb angle between the groups at a 0.05 level of significance. A standard deviation of 5.12 degrees was calculated from a pilot study using a two group t-test. A mean change of 11.75 degrees was found in the worst curves in the pilot study. nQuery 6.0 software was used to determine the sample size.

### Measurement devices

All measurements analyzed and presented in this study were taken and recorded by the research assistant, a prosthotist, who was blinded to the inclusion criteria and the first author’s definition of compliance. At baseline the following standardised measurements were taken: X–Rays, scoliometer readings, height, weight and peak flow.

**X-Rays** (with the subjects barefoot) were done pre-test (in and out the brace), at six weeks of wearing the brace 23 hours a day (in the brace) and then every six months (in and out the brace) until the day the subject started weaning out of the brace The last X-ray taken, before weaning was started, was taken after the subject had been out the brace for four hours. The research assistant saw the subjects after each X-ray and every 6-8 weeks for a brace check during the treatment period. All X-Rays were taken at the same radiology department, and at the same time of day. X-rays at pre-test and final X-ray before weaning included:

Three foot PA(Posterior Anterior), (C2 to S2) view, in standing, shielding the pelvis and exposing iliac crests for Risser sign and three foot sagittal view, C2 to S2.

X-rays done every six months only included a PA view. Measurements off the X-rays included: Cobb angle measurement; kyphotic (taken T4 to T12) and lordotic angles (taken L1 to L5); rotation of apical vertebrae (most translated and rotated vertebra) measured by the Pedriolle method (degrees) and Risser sign (bone age, seen at the iliac crest, measured 1 to 5). The Risser sign at the end of the study was confirmed on X-ray with a coned anterior posterior (more reliable than PA) view of the iliac crests [49].

**Scoliometer readings**, angle of trunk rotation (ATR) in degrees, were taken pre-test, at six weeks, thereafter every four months and just before weaning, by the research assistant. The first author also took scoliometer readings at each visit of the patient for monitoring purposes with the OSI Scoliometer (Orthopaedic System Incorporated, USA), which measures the degree of rotation of deformity of the trunk [50]. A change of three degrees or more indicates possible progression, two degrees or less indicates a variation in posture. Subjects sat on the same stool for all these measurements facing the assessor.

**Height and weight** were measured at the same intervals as above. First appointment and follow-up assessments were scheduled at the same time of day, so that height readings were as accurate as possible.

**Peak flow** (ml/s) were taken in high sitting on the same chair as above using the Mini-Wright Peak Flow meter held in the dominant hand. The subject blew three times into the meter, with a 30 second break between blows. The best result was taken and recorded in ml/s [51].

The first author assessed the subjects at baseline both subjectively and objectively for the purposes of implementing the management programme. The treatment aims in the management programme included:

•Auto-correction of the spine in a three dimensional plane, including restoration of sagittal profile

•Auto-elongation

•Deflection- Correction of body parts in the frontal plane

•Derotation- Better alignment of spine in frontal and sagittal plane

•Stabilising the corrected posture

•Ergonomy- training in active daily living, taught in sitting, standing postures

•Respiratory function and education

•Patient and family education on scoliosis theory that included:

decreasing curve progression, improving cosmesis, decreasing functional limitations, coping with treatment and deformity and psychological support.

Other aims were to improve: Coordination; equilibrium; general motor capacity; muscle endurance and strength; neuromotor control of spine and to correct side shift of the pelvis, if the pelvis was translated and to achieve stability- alignment around the sacrum, pelvis and hip joints.

### Procedure

The exercise home programme designed by the first author was given to all subjects. The principles of the home programme were explained ie. elongation, breathing, frontal and sagittal alignment. Subjects were to do the exercises 4-5 days a week, for 20-25 minutes, and to wear the brace 23 hours per day. Exercises included alignment of the spine in weight bearing postures, standing (pelvis was shifted to under the transitional point of the curvature), sitting, bridging, four point kneeling, core stability, and de-rotation techniques. Breathing exercises were done with all exercises.

A description of the exercises and a picture of their curve, (for subjects to see how to correct the curve) was included in a file which the subjects kept and brought with them each time they came to physiotherapy. Included in the file was a diary that had to be completed daily including what exercises they did and the number of hours they wore their brace. The diaries were checked at each visit by the first author and once a week by their parents. Diary contents were validated with the parents. Subjects could take the brace off for bathing and exercising, and once a week for three hours when they were going to a function.

The subject’s parents/guardians were asked to photograph the subject in a bathing costume front, back and sagittal view as a record of cosmetic improvements. The first author saw the subject after the first two weeks, checked the execution of the exercises and prescribed more exercises, as appropriate. Brace wearing difficulties were addressed and subjects taught how to sit and lie in the brace.

Thereafter subjects were reassessed by the first author once a month- when the exercises, the hours of brace wearing, comfort and fit, and the number of exercise sessions done, were checked and adjusted. If subjects were not compliant, the consequences of non- compliance were explained to them.

The English version of the Quality of Life Questionnaire, BrQ [52], was administered by the first author, after a minimum of one year of wearing the brace. The answer sheets were scored, domain subscores calculated and a total BrQ score obtained. A paedriatic psychologist administered, assessed and scored the psychological questionnaire, HSPQ or the SA92/16PF during the last year of wearing the brace. Most subjects (36) completed the HSPQ and five subjects completed the 16PF.

Monthly follow-up appointments with the first author continued until skeletal maturity was reached. Once skeletal maturity had been reached and height had been static for six months, weaning out the brace was started, which is when the study ended. The optimal weaning from a brace is not known [8]. The Risser sign at the end of the study was confirmed on X-ray with a coned anterior posterior view of the iliac crests [49]. This study took five years.

### Data analysis

Subjects were divided into two groups according to their compliance. Groups were compared with respect to Cobb angle, rotation of apical vertebrae, kyphosis and lordosis angle, scoliometer readings, peak flow, height, age, QoL and personality traits**.**

The two groups were compared at both baseline and endpoint using the student’s two- sample t- test with equal variance. The latter results were confirmed with the Welch t- test taking into account that the groups may have had different variances and Wilcoxon rank-sum test (Mann- Whitney test). The groups were compared with respect to change from baseline to endpoint using an Ancova with baseline as covariate. Also of interest were the change from baseline to end, within a group (compliant or non compliant) and testing was done using a students paired t- test. Lastly, in subjects with two curves the observation vector (thoracic cobb angle; lumbar cobb angle) was compared in the two groups using Hotelling’s T^2^-test, with univariate t- tests for thoracic Cobb angle and lumbar Cobb angle respectively, at baseline, end of treatment and change from baseline to end of treatment. Hotelling’s T^2^-test was used as in some Thoracic Type curves, there were two curves, one major and one very minor curve, therefore all subjects with two curves were compared. Testing was done at the 0,05 level of significance. Data analysis was done using: StataCorp. 2009. Stata: Release 11. Statistical Software. College Station. TX: StataCorp LP.

The individual BrQ scores of the compliant and non compliant groups were analysed and compared with a two- sample t- test with equal variance. The outcome was confirmed with both the Welch t-test and the Wilcoxon rank-sum test. The HSPQ and 16PF were analysed through scoring and interpretation as stipulated in the questionnaire HSPQ and 16PF/SA92 manuals and according to the normal hand scoring method used by the psychologist. South African norms and a sten scale were applied during interpretation of the scores according to the guidelines set out in the manuals. Sten scale scores in the two groups were compared using a two– sample t- test with equal variance. The outcome was confirmed with both the Welch t- test and the Wilcoxon rank-sum test. As a result of the personality factor traits being marginally significantly different between the compliant and non compliant groups, the groups were then compared using Pearson’s chi-square test after categorizing the trait scores into low, average and high sten scores.

## Results

Results are presented on 47 subjects as 4 dropped out early on in the study. There were three different curve types in the whole group (see Table 2 above).

**Table 2:** Curve types in the study (n = 47)

**Curve type**	**Description of curve type**	**Number of subjects**
Double major curves	Two Cobb angles: one in thoracic area (T) and one in lumbar (L) or thoracolumbar (TL) region. Similar Cobb angle size.	31 (22 were thoracic and lumbar: 9 were thoracic and thoracolumbar)
	(T + L ) or (T + TL)	
Thoracic curves	Main curve, apical vertebra in thoracic region. There could be a minor (smaller) lumbar curve.	13
Thoracolumbar curves	A single curve, apical vertebra at the thoracolumbar junction, T12 or L1.	3
	Total	47

### Baseline measures

The Cobb angle size distribution in the two groups is seen in Table 3. Cobb angles shown are those for the worst curve in a subject.

**Table 3:** The Cobb angle distribution of the worst curve (in a subject) in each group at baseline (n = 47)

**Cobb angle**	**Total**	**Compliant n (%)**	**Non compliant n (%)**
20 - 29°	12	3 (25)	9 (75)
30 - 39°	23	16 (70)	7 (30)
40 - 50°	12	7 (58)	5 (42)
	47	26 (55)	21 (45)

The two groups were well matched for all variables measured as there were no significant differences between the two groups, except for the thoracic Cobb angles (p = 0.04) and their apical rotations (p = 0.04). The number of subjects within a Risser sign are seen in Table 4.

**Table 4:** Risser sign in groups at baseline (n = 47)

**Risser sign**	**Compliant group n (%)**	**Non compliant group n (%)**	**Total**
0	2 (50)	2 (50)	4
1	4 (57)	3 (43)	7
2	8 (47)	9 (53)	17
3	10 (59)	7 (41)	17
4	2 (100)	0 (0)	2
Total	26	21	47

The Lonstein Progression Risk Factor as well as the percentage of in-brace correction a subject had at baseline are shown in Table 5. There was no significant difference between the two groups for progression risk of the curvatures and the in-brace corrections.

**Table 5:** Progression risk and in-brace correction for groups at baseline (n = 47)

**Measure**	**Group**	**n (%)**	**Mean (±SD)**	**p value**
Progression risk	Compliant (n = 26)	26 (100)	78.46 (±28.1)	0.34
(percentage)	Non compliant (n = 21)	21 (100)	70.48 (±28.94)	
In Brace Correction	Compliant	21 (81)	44.52 (±16.95)	0.15
(percentage)	Non compliant	16 (76)	35.75 (±18.81)	

There were no significant differences between the “Double Major curves” and “Thoracic curves” in the two groups, at baseline. The two curves within the “Double Major curves”, those with lumbar curves (T + L) and those with thoracolumbar curves (T + TL) could not be separated as the numbers were too low in the compliant and non -compliant groups.

### Measures after the intervention

The Cobb angle of the worst curve per subject is illustrated in Table 6.

**Table 6:** The Cobb angle distribution of the worst curve (in a subject) in each group at the end of the study (n = 47)

**Cobb angle**	**Total**		**Compliant n (%)**		**Non Compliant n (%)**	
	**(@ Baseline)**	**End**	**(@ Baseline)**	**End (%)**	**(@ Baseline)**	**End (%)**
< 20°	(0)	5	(0)	**5** (100)	(0)	**0** (0)
20 - 29°	(12)	19	(3)	**14** (74)	(9)	**5** (26)
30 - 39°	(23)	14	(16)	**6** (43)	(7)	**8** (57)
40 - 50°	(12)	9	(7)	**1** (11)	(5)	**8** (89)
Total	(47)	47	(26)	**26**	(21)	**21**

(Number at baseline of study is in the first set of brackets).

Five subjects dropped below 20° in the compliant group and the number of subjects in the larger Cobb angle ranges decreased, with only one subject in the 40-50° range reducing the risk of surgery, which is indicated at 50° [53,54]**.** In the non- compliant group the numbers in the larger Cobb angle ranges, increased with eight subjects at 40-50° so increasing the indication of surgery. Overall, the need for surgery was decreased as the total number of larger curves was decreased. In the non- compliant group three subjects did have surgery as their curvatures were large (Cobb angle > 45°) and they had not yet reached skeletal maturity.

There were significantly more brace wearing hours in the compliant group as can be seen in Table 7.

**Table 7:** Brace hours and exercise sessions at end of study in compliant and non compliant groups (n = 47)

**Measure**	**Group**	**n (%)**	**Mean (±SD)**	p value*
Average hours of brace	Compliant (n = 26)	26 (100)	21.5 (±1.17)	**<0.0001**
Wearing per day (hours)	Non compliant (n = 21)	21 (100)	12.19 (±7.05)	
Average exercise	Compliant	26 (100)	3.92 (±0.63)	**<0.0001**
Sessions per week	Non compliant	21 (100)	1.71 (±1.06)	

(bold font = significant p values).

All Cobb angles were significantly lower in the compliant group with the worst Cobb angle (25.38° ± 8.3) being significantly lower than (36.71° ± 9.3) (non compliant) (p = 0.0001). The associated worst apical rotation was also significantly lower in the compliant group (11.54° ± 7.9) compared to (17.95° ± 9) (p = 0.01) (non- compliant). The kyphosis Cobb angle was significantly higher in the compliant group (34.33° ± 6.4) and well within normal limits compared to (21.5° ± 6.9) (non- compliant), which was below normal limits, being 25-50° [55]. The number of subjects with lordosis and kyphosis measurements was however small. The scoliometer readings in the lumbar area were significantly lower in the compliant group (1.38° ± 2.5) compared to (4.67° ± 4) (non- compliant) (p = 0.001).

In the whole group of Double Major curves (31) the thoracic and lumbar Cobb angles, with their apical rotations, were significantly smaller in size in the compliant group. In the Thoracic Curve type (13), the thoracic Cobb angles (p = 0.03) and the apical rotations (p = 0.05) were significantly smaller in the compliant group. Those with two curves had significantly smaller Cobb angles (p = 0.005) and apical rotations (p = 0.03) in the compliant group.

During the study, the age of menarche was not significantly different in the two groups. Age of menarchy in the compliant group was 12.9 (±0.8) years and non- compliant group 13.06 (±1.37) (p = 0.83). Skeletal maturity, was between 3.46 (±0.5) (non compliant group) to 4.03 (±0.6) (compliant group) years after menarche and not significantly different. Age at menarche data could not be obtained on all subjects and therefore only 18 subjects were included in this analysis. The subjects’ heights were static 3.46 - 4.03 years after menarche, when weaning was started.

### Change in measures, baseline to endpoint

The change in measurements during the study, from baseline to endpoint is presented in Table 8.

**Table 8:** Change in study data, baseline to end (n = 47)

**Measure**	**Group**	**n (%)**	**Mean (±SD)**	**p value**
All Thoracic Cobb	Compliant (n = 26)	25 (96)	8.96 (±6.10)	**<0.0001**
angles (degrees)	Non compliant (n = 21)	21 (100)	- 5.81 (±6.87)	
All Lumbar Cobb	Compliant	19 (73)	7.11 (±4.99)	**<0.0001**
angles (degrees)	Non compliant	19 (90)	- 3.11 (±4.98)	
Worst Cobb angle	Compliant	26 (100)	10.19 (±5.46)	**<0.0001**
(degrees)	Non compliant	21 (100)	- 5.52 (±4.31)	
Worst Apical	Compliant	26 (100)	7.42 (±7.15)	**<0.0001**
Rotation (degrees)	Non compliant	21 (100)	- 3.67 (±6.51)	
All (Thoracic) rotation	Compliant	26 (100)	4.92 (±6.94)	**0.001**
Apical Vertebra	Non compliant	21 (100)	- 3.81 (±7.23)	
All (Lumbar) rotation	Compliant	26 (100)	3.65 (±6.09)	**0.0005**
Apical Vertebra	Non compliant	21 (100)	- 1.05 (±3.75)	
Kyphosis angle	Compliant	5 (19)	3.60 (±6.88)	0.24
(degrees)	Non compliant	2 (10)	- 1.00 (±8.49)	
Lordosis angle	Compliant	4 (15)	1.50 (±8.70)	0.73
(degrees)	Non compliant	2 (10)	- 3.00 (±25.46)	
Thoracic, scoliometer	Compliant	26 (100)	2.46 (±2.34)	**0.04**
Reading (degrees)	Non compliant	21 (100)	0.33 (±3.99)	
Lumbar, scoliometer	Compliant	26 (100)	3.65 (±3.61)	**<0.0001**
Reading (degrees)	Non compliant	21 (100)	- 0.19 (±2.77)	
Peak flow	Compliant	26 (100)	63.31 (±35.39)	**0.04**
(l/min)	Non compliant	20 (95)	31.15 (±49.92)	
Height	Compliant	26 (100)	3.64 (±2.80)	0.43
(meters)	Non compliant	21 (100)	3.61 (±5.13)	
Time spent in brace	Compliant	26 (100)	2.80 (±1.18)	**0.0009**
(years)	Non compliant	21 (100)	2.27 (±1.00)	

(bold font = significant p values).

In the compliant group the mean thoracic Cobb angle, lumbar Cobb angle, worst Cobb angle, all apical rotations and scoliometer readings, all improved, whereas in the non -compliant group these measures all deteriorated (p < 0.0001). The worst Cobb angles in the compliant group improved 10.19° (±5.5) and deteriorated 5.52° (±4.3) in the non- compliant group (p < 0.0001). The worst curve apical vertebral rotation in the compliant group improved 7.42°(±7.15) and deteriorated 3.67° (±6.51)(non- compliant group)(p < 0.0001). Scoliometer readings (taken in thoracic and lumbar regions) show that the compliant group significantly improved cosmetically, compared to the non- compliant group (lumbar region, p < 0.0001: thoracic region, p = 0.04). The compliant group improved their peak flow significantly, by a mean of 20 percent, compared to an improvement by nine percent (non -compliant group)(p = 0.04). The compliant group wore the brace for a significantly longer period (2.8 ± 1.18 years) than the non compliant group (2.27 ± 1 years), in which three subjects had surgery before skeletal maturity was reached (p = 0.0009).

Similarly there was a significant improvement in the Double Major curve types, mean thoracic and lumbar Cobb angles, and their respective apical rotations in the compliant group and deterioration in the non- compliant group. Thoracic Curve type change is illustrated in Table 9.

**Table 9:** Change in data, baseline to end, in Thoracic Type Curves in compliant and non compliant groups (n = 47)

**Measure**	**Group**	**n (%)**	**Mean (±SD)**	**p value**
All Thoracic Cobb	Compliant (n = 26)	9 (35)	12.56 (±7.07)	**0.004**
angles (degrees)	Non compliant (n = 21)	4 (19)	-6.00 (±6.68)	
All (Thoracic) rotation	Compliant	9 (35)	8.67 (±9.07)	**0.01**
Apical Vertebra	Non compliant	4 (19)	-6.25 (±7.50)	

(bold font = significant p values).

There was a significant improvement in thoracic Cobb angle change of 12.56° in the compliant group compared to the non- compliant group which deteriorated six degrees in Thoracic type curves. Apical rotation improved in the compliant group and deteriorated in the non- compliant group.

At the end of the study, in subjects with two curves, the mean thoracic and lumbar Cobb angles and their apical rotations, improved significantly in the compliant group and deteriorated in the non- compliant group. Hotelling’s T^2^ p value for the vector (thoracic Cobb angle; lumbar Cobb angle) showed a significant improvement in the compliant group for mean Cobb angles (p < 0.0001) and apical rotations (p < 0.0001).

The difference between baseline and endpoint measurements within groups was compared, and within the compliant group, the mean Cobb angles, apical rotations and worst Cobb angles all improved significantly and decreased in size whereas these measures all deteriorated significantly and increased in size in the non- compliant group.

### Brace questionnaire

The compliant group had significantly higher scores illustrating a significantly better quality of life, better self esteem and general health (Table 10). In addition they had significantly more energy, vitality (p = 0.0004), were more satisfied with their body image, had better physical functioning in the brace during normal daily activities and better school activity. The compliant group’s emotional functioning was better, they were happier, believed the brace to be beneficial and had a significantly better health perception (p = 0.04). The non -compliant group had significantly more pain with wearing the brace and saw themselves as being more sickly. In the Social Function domain, however the scores were marginally significantly higher in the compliant group, indicating subjects could socialise with their friends, did not feel different from their peers and had few problems with their family**.**

**Table 10:** Brace questionnaire results (n = 45)

**Measure**	**Group**	**n (%)**	**Mean (±SD)**	**p value**
Total brace	Compliant (n = 26)	26 (100)	81.65 (±10.65)	**0.001**
Questionnaire score	Non compliant (n = 19)	19 (100)	69.52 (±12.25)	
**Domains**				
General health	Compliant	26 ( 100)	8.12 (±1.73)	**0.04**
Perception	Non compliant	19 (100)	6.89 (±2.26)	
Physical function	Compliant	26 (100)	29.85 (±3.18)	**0.003**
	Non Compliant	19 (100)	25.95 (±5.05)	
Emotional function	Compliant	26 (100)	18.58 (±3.84)	**0.05**
	Non compliant	19 (100)	15.89 (±5.37)	
Self esteem	Compliant	26 (100)	7.85 (±1.38)	**0.003**
	Non compliant	19 (100)	6.26 (±2.02)	
Vitality	Compliant	26 (100)	7.92 (±1.26)	**0.0004**
	Non compliant	19 (100)	6.00 (±2.08)	
School activity	Compliant	26 (100)	13.42 (±1.77)	**0.03**
	Non compliant	19 (100)	12.11 (±2.23)	
Bodily pain	Compliant	26 (100)	25.57 (±3.75)	**0.01**
	Non compliant	19 (100)	22.52 (±3.96)	
Social function	Compliant	26 (100)	26.34 (±5.15)	0.07
	Non compliant	19 (100)	23.16 (±6.42)	

(bold font = significant p values).

### High school personality questionnaire and 16 PF

Questionnaires were completed by 23 subjects in the compliant group and 18 in the non -compliant group. There was only a marginally significant difference between compliant and non compliant groups in Factors C, E, I and Q₄.

**Factor C** in the compliant group had a marginally significantly higher score (p = 0.098) indicating that the compliant group was marginally more ‘emotionally mature, stable and realistic’ than the non- compliant group, who were more ‘emotionally immature and unstable’, but the difference is minimal.

**Factor E** in the compliant group had a marginally significantly lower score (p = 0.07) reflecting that the compliant group was marginally more ‘obedient, mild and dependant’ than the non- compliant group, who were more ‘assertive, aggressive, rebellious, dominance’ score.

**Factor I** in the compliant group had a marginally significantly higher score (p = 0.06) indicating that the compliant group was marginally more ‘tender minded, sensitive, protected’ than the non -compliant group, who were more ‘practical and tough minded’ .

**Factor Q₄** in the compliant group had a marginally significantly lower score (p = 0.099) showing that the compliant group was marginally more ‘relaxed, composed’ than the non- compliant group, who were more ‘tense, driven, irritable’, but the difference is minimal.

As a result of the personality factor traits being marginally significantly different between the compliant and non compliant groups, the groups were then compared after categorizing the trait scores as:

Low sten scores (1, 2 & 3) = Category 1

Average sten scores (4, 5, 6, 7) = Category 2

High sten values (8, 9 & 10) = Category 3

The only significant difference was found in Factor C (Table 11). There was a significant difference between the compliant and non- compliant groups (p = 0.03) for Factor C only, with the compliant group having significantly more subjects in the average and high sten scores, compared to the non- compliant group where most of the subjects were in the low and average sten score category. The compliant group was significantly more emotionally mature, stable and realistic (p = 0.03).

**Table 11:** Factor C, category differences in compliant and non compliant groups (n = 41)

**Factor category**	**Compliant n (%)**	**Non compliant n (%)**	**Total**	**p value**
1 Low score	0 (0.00%)	3 (16.67%)	3 (7.32%)	**0.03**
2 Average Score	14 (60.87%)	13 (72.22%)	27 (65.85%)	
3 High score	9 (39.13%)	2 (11.11%)	11 (26.83%)	
Total	23 (100.00%)	18 (100.00%)	41 (100.00%)	

(bold font = significant p values).

## Discussion

The results of this study indicate that compliance to a specific exercise programme and wearing the RSC brace can improve curvatures and signs and symptoms of AIS. The natural history of scoliosis was altered in the compliant subjects with non-compliance resulting in significant progression of the curvatures. Some studies have shown that conservative management of bracing and exercise has no effect on the natural history of scoliosis while others have shown that conservative management is effective [4,23], but compliance was not reported on in these studies. The importance of compliance is supported by Landauer et al. [24] and Weinstein et al. [23], and should be monitored in future studies [23].

The risk of progression of the curvatures in both groups of subjects was high, 70-78 percent. The mean in-brace correction was 44.5% in the compliant group and 35.8% in the non -compliant group, which was not statistically different with similar in-brace corrections being reported [56,57]. Compliance and initial correction effect in the brace are the two most important variables associated with good brace outcomes [5,24,25,58]. The reason for the good in-brace correction in this study is probably the good three dimensional design of the RSC brace [59].

A study by Landauer et al. [24] has similar results to this study. They advised full time Cheneau bracing and weaned the subjects at Risser 5 over a six month period. They did not state how many hours the brace was actually worn, however, they did use a compliance score. The final overall outcome of their study, including all subjects, was that the thoracic curve improved three degrees only, not a successful result. However, when the subjects were divided into compliant and non compliant groups, then the study was very successful, as high early in-brace correction (40% or more) and good compliance achieved a correction of seven degrees in the Cobb angle. Low early correction (less than 40%) and good compliance resulted in stabilisation of the curvatures. Poor compliance with a high or a low initial correction resulted in progression of the Cobb angle [24] therefore emphasising the importance of evaluating compliance which is more important than in-brace correction.

The compliant group in this study had improved Cobb angles, angle of vertebral rotation and angle of trunk rotation (ATR), by wearing the brace 21.5 hours per day. Wearing the brace for 12 hours in the non- compliant group resulted in progression of the curvatures. Many Cheneau brace studies do not mention the number of hours the brace was worn [17,54,60,61] but good brace compliance has been shown by a number of studies to have good outcomes [21-24,62-65]. Recording the brace wearing hours was one of the challenges of this study. Using a compliometer or thermobrace, not currently used in South Africa, would have been more accurate in measuring actual brace wearing hours. Subjects’ diaries were checked frequently by the first author and parents for the record of brace hours. A study by Takemitsu et al. [66] showed that patients complied with 75 percent of a prescribed routine and on average over-reported their hours of brace wear to their physicians. The actual brace hours were measured using a compliance monitor. A study by Donzelli et al. [67] in which they used a temperature sensor (Thermobrace) showed compliance to be higher than previously reported with brace prescription being 16-23 hours per day and more than half the patients had 90 percent compliance.

The exercise programme was completed 3.9 times a week by the compliant group and 1.7 times a week by the non -compliant group This prescription of four to five times a week for 20-25 minutes is similar to the Schroth method [68], Dobosiewicz method [69], and SEAS and Side Shift method [26].

The Cobb angles were significantly reduced in the compliant group with the worst/major Cobb angle significantly improving (10.19 degrees), thus the natural history of AIS was altered. In the non -compliant group there was significant progression of the curvatures (5.5 degrees), following the natural history of scoliosis and three progressed to surgery. Supporting this study is a small study by Wood [59] on 23 subjects, with Cobb angles greater than 30 degrees, using the Cheneau brace over a four year period with a progression risk of 68 percent. Compliance was not measured in the Wood study nor was the physiotherapy described. Wood [59] showed that the major Cobb angle improved a mean of 13.2 degrees and the minor Cobb angle improved eight degrees at the end of brace wearing. Physiotherapy has been shown to have favourable outcomes in scoliosis patients and Rigo claims that physiotherapy can improve the actions of the Cheneau brace, by making the curve more flexible and preventing muscle atrophy [17,70]. Cinnella et al. [71] using the Cheneau brace showed a 23 percent correction in Cobb angle at the end of a mean treatment period of 4.5 years, and after five years the correction was 15 percent but compliance was not monitored. Other studies on the Cheneau and RSC braces only show stabilisation of curvatures and reduction in children requiring surgery [6,14,64,65,72-74].

Kyphosis angles improved in the compliant group and deteriorated in the non- compliant group, and therefore the sagittal profile improved in the compliant group. The compliant group is similar to several studies that show normalisation of kyphosis and lordosis with the Cheneau brace [13,17,60]. In many Cheneau brace studies however, the sagittal profile is not mentioned, [24,56,59,61,65,71]. The RSC brace allows sagittal normalisation because of its physiological profile in the sagittal plane and every trunk section is aligned to allow a normal sagittal profile [17,75].

The angle of axial rotation predicts the incidence of progression of a curve better than the size of the Cobb angle [76]. In this study all apical vertebra were significantly reduced in rotation in the compliant group p < 0.0001 in the worst/major curves which is similar to the study by Wood [59]. The RSC and the Cheneau brace has also been shown to improve the wedge deformity of the apical vertebra, by over 50% in some cases [77,78].

Scoliometer readings improved significantly in the compliant group. Similarly Kinel et al. [63,79] showed that girls with AIS, wearing a Cheneau brace, revealed less clinical deformity than a group of non- treated girls with similar radiological deformities. Other studies using the Cheneau brace have shown cosmetic improvements in the deformity [13,17,59,60,80].

The peak flow changes in the compliant group improved significantly (by a mean of 20 percent) more than the non -compliant group (by nine percent). The Dobomed method has also been shown to improve exercise efficiency significantly using ergospirometry [69,81]. Other studies using outpatient or in-patient Schroth method have shown significant improvements in vital capacity (VC) [82-85]. Dos Santos Alves et al. [86] showed that aerobic exercises three times a week for an hour, over a period of four months, resulted in a significant improvement in FVC, FEV1, inspiratory capacity, expiratory reserve volume and in respiratory muscle strength [87]. Spirometry is the method of choice to identify any changes in the course of a respiratory disease [88] although a Mini Wright peak flow meter was used here to measure FEV1, as it is simple, portable, reproducible and practical to use clinically [89,90].

Peak height was reached between 3.46 (±0.5) to 4.03(±0.6) years after menarchy. Weaning the subjects out of full time bracing, at the end of the study, occurred once height had been static for six months and as close to Risser 5 as possible. Weaning out the brace in the compliant group of this study started at the mean age of 17.14 (±0.6) years, which is later than most studies, in order to prevent progression, previously reported and postural collapse [12,91]. The fact that peak bone mineralization and peak muscle strength occurs at 25, and peak ligamentous stability occurs in the early 20’s [92], was considered when deciding on weaning. Bracing is sometimes considered ineffective, when actually the subjects have just been weaned out too early or too quickly [93]. The optimal weaning process is not known and has not been standardised [8,12] and therefore was not included in this study. Skeletal maturity measures are not accurate enough to predict spinal growth potential in AIS [94].

One subject stopped wearing the brace in the non- compliant group**.** Psychosocial and body image disturbance are less marked in patients with good social and family functioning, as well as patients who exercise regularly [40] and this is similar to the compliant group in this study. The compliant group had larger Cobb angles than the non compliant group at baseline, therefor the severity of the scoliosis as measured by Cobb angle was not related to a poorer quality of life. Other Cheneau brace studies have shown the brace negatively affects quality of life [95-97]. Quality of life issues may be related to psychosocial coping mechanisms more than the physical deformity and its consequences. Support for AIS patients in group or individual sessions prevents psychosocial impairment, body image disturbances and should be included in holistic management plans [34,40]. Programmes to address personal, group and family issues may improve QoL, promoting compliance [43]. In this study emotional function was lower in the non- compliant group, and subjects did not believe that the brace was beneficial, had low self esteem and low social function. Lindeman and Behm [98], showed that non- compliant girls did not expect to succeed in dealing with scoliosis, they were anxious about possible failure, had low self- esteem and did not seek social support.

Non compliance to the intervention resulted in progression in curvatures. These subjects had a poorer quality of life and seemed to need psychosocial support to improve compliance and therefore treatment outcomes. This should be included as part of a management programme, as subjects do not generally seek help. Continuous monitoring of stress and QoL needs to be done, which will allow modification of the treatment and maintain good compliance [42] with regular consultations with a psychologist and family counselling.

The personality trait questionnaire revealed, that the compliant group was more emotionally mature, stable and realistic than the non- compliant group, which was more emotionally immature and assertive (Factor C). Higher scores in Factor C (compliant group) reveal emotional stability, control, and high ego strength [47]. These patients appear calm, unruffled, behave in an adult and rational way, they are realistic, constant in interests, responsible, distinguish between emotional needs and reality, and adjust to facts. High Factor C scores also correlate with positive family relationships and leadership [45]. Lower Factor C scores, in the non- compliant group, indicated emotional instability and low ego strength [45]. They reveal an inability to control their emotions, impulses and to find satisfying and realistic ways of expressing them. They are easily angered, are more frequently dissatisfied with their family and school, find it difficult to restrain themselves and are discouraged by their inability to meet good standards of behaviour. They are easily perturbed, confused, changeable in attitudes and interests. They evade responsibility, give up easily, tend to worry a lot, have irrational fears and get into fights and problem situations. They can experience severe adjustment problems if subjected to regimentation and stress [45] with bracing being stressful [35]. These factors may explain their lack of compliance.

Psychological support during this adjustment to bracing and exercise phase as well as during the treatment therefore seems to be essential. The results of the personality questionnaire compare well with the results of the BQ, which showed that the non- compliant subjects had a poorer QoL.

Other traits from the questionnaire, revealed marginally significant differences in that the compliant group was more obedient, mild and dependant (Factor E), tender minded, sensitive, protected (Factor I) relaxed, and composed (Factor Q₄). The non- compliant group was more aggressive, rebellious, dominant (Factor E), practical tough- minded and tense (Factor I) driven, irritable, and frustrated (Factor Q₄). In Factor E, lower scores, in the compliant group, show traits that are more accommodating, more compliant and easily influenced [45]. High scores in Factor E seen in the non- compliant group, show traits of stubbornness, and are headstrong, arrogant and disobedient.

This study has determined the personality traits of compliant and non- compliant subjects and these traits can be used to predict compliance of a subject. Should a patient be predicted to be non -compliant using the HSPQ, at the beginning of treatment, then appropriate interventions, such as regular individual and family counselling could be implemented at the beginning of the programme in an attempt to improve compliance. The up to date personality questionnaire now recommended by the editors of the HSPQ is the “16PF Adolescent Personality Questionnaire”, by Scheurger [99], for 11-22 year olds.

## Conclusions

Good compliance to a conservative treatment programme of the RSC brace and a specific exercise regime resulted in a significant improvement in curvatures, whereas poor compliance resulted in progression of curvatures. Predicting compliance using personality traits at the start of a conservative treatment programme could indicate what interventions are required to improve compliance. Further studies are required to determine the long term effects of this conservative treatment programme.

## Abbreviations

AIS: Adolescent idiopathic scoliosis; ATR: Angle of trunk rotation; BrQ: Brace questionnaire; DoboMed: The dobosiewicz method; FVC: Forced vital capacity; HRQoL: Health related quality of life; HSPQ: High school personality questionnaire; IS: Idiopathic scoliosis; l/min: Litres per minute; 16 PF: Sixteen personality factor questionnaire; PA: Posterior anterior; QoL: Quality of life; RCT: Randomised controlled trial; RSC Brace: Rigo System Cheneau brace; SEAS: Scientific exercises approach to scoliosis; SIR: Scoliosis intensive rehabilitation program; SOSORT: Society on Scoliosis Orthopaedic and Rehabilitation Treatment; VC: Vital capacity.

## Competing interests

The authors declare that we have no competing interests.

## Authors’ contributions

LR designed the study, applied exercise programmes, administered the Brace Questionnaires, collected the data, analysed and interpreted the data. LR drafted the manuscript and revised it critically for important intellectual content. AS assisted with study design, drafting of manuscript and supervision of the study. JP assisted with supervision and drafting of manuscript. All authors give final approval of the version to be published.

## Authors’ information

LR is a physiotherapist in private practice with a PhD degree from the University of the Witwatersrand Johannesburg South Africa.

AS is an Associate Professor with a PhD in the Department of Physiotherapy, School of Therapeuitc Sciences, Faculty of Health Sciences University of the Witwatersrand Johannesburg South Africa.

JP is an Associate Professor with a PhD in the Department of Physiotherapy, School of Therapeutic Sciences, Faculty of Health Sciences University of the Witwatersrand Johannesburg South Africa.
